# Severe Bleeding Diathesis in Siblings with Platelet Dysfunction due to a Novel Nonsense *RASGRP2* Mutation

**DOI:** 10.1055/s-0040-1718910

**Published:** 2020-12-25

**Authors:** Julia Körholz, Nadja Lucas, Franziska Boiti, Karina Althaus, Oliver Tiebel, Mingyan Fang, Reinhard Berner, Min Ae Lee-Kirsch, Ralf Knöfler

**Affiliations:** 1 Department of Pediatrics, Medizinische Fakultät Carl Gustav Carus, Technische Universität Dresden, Dresden, Germany; 2 Institute for Transfusion Medicine, University Hospital Tübingen, University of Tübingen, Tübingen, Germany; 3 Institute for Clinical Chemistry and Laboratory Medicine, Medizinische Fakultät Carl Gustav Carus, Technische Universität Dresden, Dresden, Germany; 4 BGI-Shenzhen and China National GeneBank, Shenzhen, China

**Keywords:** bleeding diathesis, platelet dysfunction, *RASGRP2*

## Abstract

Next-generation sequencing is increasingly applied during the diagnostic work-up of patients with bleeding diathesis and has facilitated the diagnosis of rare bleeding disorders such as inherited platelet function disorders. Mutations in RAS guanyl releasing protein 2 (RasGRP2), also known as calcium- and diacylglycerol-regulated guanine nucleotide exchange factor I (CalDAG-GEFI), underlie a recently described platelet signal transduction abnormality. Here we present the case of a consanguineous family originating from Afghanistan with two siblings affected by recurrent severe mucocutaneous bleedings. Platelet function testing demonstrated a marked reduction of aggregation induced by collagen and adenosine diphosphate. Whole exome sequencing revealed a novel homozygous nonsense *RASGRP2* mutation segregating with the bleeding disorder in the family.

## Introduction

During the last decade, advances in human genetics have enormously broadened our knowledge of the pathogenesis of inherited platelet function disorders (IPFDs). 1 Among them are dysfunctions in platelet activation pathways caused by *RASGRP2* mutations encoding the RasGRP2 protein, also known as calcium- and diacylglycerol-regulated guanine nucleotide exchange factor I (CalDAG-GEFI). 1 2 RasGRP2 is a guanine exchange factor (GEF) that facilitates substrate dissociation in guanosine triphosphatases (GTPases), termed Ras. These GTPases are important regulators in cell signaling. The RasGRP family consists of four members with different tissue expression, of which RasGRP2 is the only member expressed in platelets. RasGRP2 is also referred to as CalDAG-GEFI (calcium and diacylglycerol-regulated guanine exchange factor I). The protein is essential for αIIβ3 integrin activation and fibrinogen binding during platelet aggregation. The associated bleeding disorder has been referred to as bleeding disorder platelet-type 18 (BDPLT18). 3 Altogether, more than 20 human disease-causing genetic *RASGRP2* variants have been reported up to today. 3 4

In the first description of mutant RasGRP2, Canault et al reported an abolished Rap1 activation in patient platelets upon stimulation, indicating defective platelet aggregation. 5 In contrast, other platelet activation mechanisms including protein kinase C and adenosine diphosphate (ADP) dependent pathways were not affected by *RASGRP2* mutations. In general, glycoprotein receptor expression is usually not impaired, whereas thrombus formation and platelet spreading have been demonstrated to be impaired in murine RasGRP2 deficiency. 3 5 6 Consequently, typical findings of platelet aggregation testing in patients include an absent or reduced aggregation potential in response to low concentrations of the platelet agonists ADP and collagen. 7 Clinically, affected individuals exhibit a severe bleeding tendency, especially at young age. Heterozygous mutation carriers are asymptomatic, although their platelets may display a reduced adhesion under flow and spreading in vitro. 5

Here we describe two sisters from a consanguineous family who presented with a childhood-onset severe bleeding disorder due to a novel homozygous *RASGRP2* mutation.

## Materials and Methods

### Study Approval

Written informed consent was obtained by all participating members of the family or their legal guardians. Patients were enrolled in an ongoing study on rare diseases approved by the Ethics Committee of the Medical Faculty, Technische Universität Dresden.

### Whole Exome Sequencing

Genomic DNA was extracted from blood using the Qiamp DNA Blood Mini Kit (Qiagen). Constructed exome libraries were subjected to Illumina Hiseq4000/Xten (Illumina) 150-bp paired-end sequencing with an average read depth of 134.5X. SOAPnuke was used to remove adapter sequences, low-quality reads, and N reads 1. Sequences were mapped to the human reference genome (GRCh37 / UCSC hg19) using the Burrows–Wheeler Algorithm (BWA-MEM, version 0.7.10) 2. GATK Haplotype Caller (version 3.3) 3 was used for single nucleotide variants and insertions/deletions (InDels) calling and Ensembl VEP (Variant Effect Predictor) 4 for annotation.

### Sanger Sequencing

The mutation of the *RASGRP2* (NM_001098671) gene was amplified by polymerase chain reaction using gene-specific primers (Eurofins MWG Operon; RASGRP2_for-CTTTGACCCTTCGGAGTCAG, RASGRP2_rev-CCTGAGCTCTGGGATAAGGA) and sequenced in both directions using the BigDye Terminator v1.1 Cycle Sequencing Kit (Applied Biosystems) on a 3130xl Genetic Analyzer (Applied Biosystems). Data were analyzed using the Vector NTI Software (Life Technologies).

### Platelet Function

Aggregometric testing, using an APACT aggregometer (Labor BioMedical Technologies GmbH) and a Chrono-Log Lumi-aggregometer (Chrono-Log Corporation), and flow cytometry (FACSLyric, Becton Dickinson) were performed by standard methods.

## Case Presentation

**Fig. 1:**
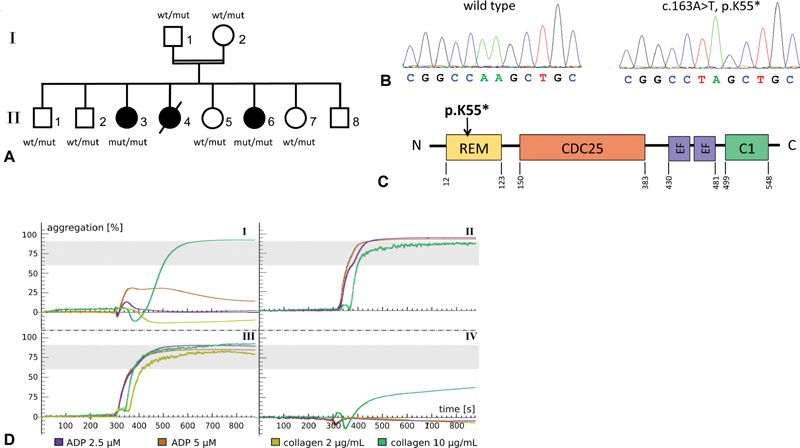
Genetic and functional findings. ( **A** ) Pedigree of family with clinically affected individuals colored in black. ( **B** ) Electropherograms of wild type and mutant sequences depicting the homozygous *RASGRP* 2 mutation. ( **C** ) Protein structure of RasGRP2 and location of the here described mutation. ( **D** ) Aggregometric analysis of (I) patient 1 exemplary for a homozygous *RASGRP2* mutant phenotype, (II) a heterozygous carrier, (III) a normal control, and (IV) a patient with Glanzmann's thrombasthenia. In individuals affected by a homozygous *RASGRP2* mutation, platelet aggregation is impaired by standard concentrations of the inductors ADP and collagen but is normal at a markedly elevated collagen concentration of 10 µg/mL. The reference range of maximum aggregation for both concentrations of collagen and ADP is between 60 and 90%, as indicated by the area colored in gray. ADP, adenosine diphosphate; wt, wild type; mut, mutant.

Index patient (patient 1) is the daughter of consanguineous healthy parents originating from Afghanistan who presented at 7 years of age at our hospital (II.6, Fig. 1A ). She suffered from severe episodes of epistaxis as the main bleeding symptom since the first year of life. Bleeding occurred several times a month, sometimes requiring admission to the hospital for treatment. The parents also reported prolonged bleedings after minimal trauma as well as the need for blood transfusions in the context of an abdominal surgery for unknown reasons in Iran. Menarche occurred at the age of 10 years. From the second menstruation onward, she experienced heavy bleedings requiring repeated erythrocyte transfusions. Ancillary findings included lichen planus and hepatic echinococcosis.

Patient 2 is the older sister of patient 1 (II.3, Fig. 1A ) who was first diagnosed with a platelet function defect at the age of 15 years. Like her sister, she presented with recurrent nose bleeds several times a month. She further experienced massive hypermenorrhea with continuous uterus bleeding for up to 15 days. Bleeding intensity was ameliorated by treatment with an oral contraceptive. She also exhibited pronounced bleedings after minimal trauma and recurring mucosal bleedings from brushing her teeth. In addition, she was treated for nephrolithiasis, which caused macrohematuria.

Both patients were treated orally with tranexamic acid to prevent spontaneous bleedings and with iron supplementation to prevent clinically relevant anemia. However, due to incompliance, both patients continued to present with severe bleeding episodes as well as anemia that required erythrocyte transfusions on several occasions. As a consequence, we decided to treat the menorrhagia by hormone-induced anovulation.

A third sister (II.4, Fig. 1A ) who died at the age of 11 years from an uncontrolled mucosal bleeding was likely also affected by the same bleeding disorder.

By extended coagulation diagnostics thrombocytopenia, plasmatic coagulation disorders and von Willebrand syndrome were excluded. Assessment of platelet function included aggregometry in platelet-rich plasma (PRP), aggregometry and adenosine triphosphate (ATP) release by lumi-aggregometry in citrated whole blood, and flow cytometry. Aggregation in PRP was induced with ADP (2.5 and 5µM), arachidonic acid (1.64 mM), collagen (2 and 10 µg/mL), and ristocetin (0.5 and 1.5 mg/mL). In PRP, both patients showed an impaired aggregation in response to both concentrations of ADP and to the standard concentration of collagen (index patient shown in Fig. 1D ). In contrast, the response to collagen was normal at the high concentration for both patients ( Fig. 1D ). Activation with arachidonic acid and ristocetin as well showed normal aggregation. Lumi-aggregometry was performed with ADP at 20 and 30 µM, arachidonic acid at 0.5 and 1 mM, collagen at 1, 2, and 4 µg/mL, ristocetin at 1.0 mg/mL, and thrombin at 0.5 U/mL (with thrombin as determination of ATP release). In contrast to PRP, platelets from both patients in whole blood did not respond to ADP and collagen even at higher concentrations ( Supplementary Fig. S1 ). The response was normal to arachidonic acid, ristocetin, and thrombin. Healthy siblings and parents showed normal aggregation in lumi-aggregometry with all agonists used. By flow cytometry, Glanzmann thrombasthenia (CD41a), Bernard–Soulier's syndrome (CD42a, CD42b), and α- and δ-storage pool deficiency (CD62P, CD63, mepacrine uptake) were excluded.

Genetic investigation by panel sequencing was initiated, and mutations in *P2Y12* , *TBXA2R* , *PTGS1* , *ITGA2B* , *ITGB3* , and *NBEAL2* , as well as the *ANKRD26* promotor region were excluded. Finally, whole exome sequencing followed by data analysis assuming an autosomal recessive mode of inheritance revealed a homozygous *RASGRP2* mutation (NM_001098671:c.163A > T; p.Lys55Ter, p.K55*) in both the affected sisters ( Fig. 1C ). The mutation is located in the Ras exchange motif (REM) domain of the protein and predicted to cause a truncation of the C-terminal end, consistent with a loss of function. In line with this, a similar truncating mutation, p.F497fs*22, was previously shown to abrogate *RASGRP2* function. 7 However, this mutation has not previously been described in patients with bleeding disorder and has not been annotated in the ENSEMBL or gnomAD databases. Sanger sequencing confirmed full segregation of the mutation with the disease phenotype ( Fig. 1A ). Exome analysis did not reveal any evidence for segregating pathogenic variants in the *FERMT3* gene, which has recently been linked to platelet dysfunction. 8

## Discussion

This case report describes an inherited platelet function disorder due to a novel *RASGRP2* mutation as the cause of a severe bleeding tendency beginning in childhood. It illustrates the relevance of early diagnosis to provide optimal treatment and counseling. For this purpose, national and international guidelines recommend a step-by-step diagnostic work-up for assessment of coagulation function. 9 10 Accordingly, after exclusion of more common causes of abnormal bleeding such as thrombocytopenia and Von Willebrand's disease, platelet function testing should be performed. The recommended diagnostic workflow includes platelet function testing with various agonists. While aggregometry in PRP represents an established diagnostic method, it remains difficult to standardize. 10 11 The discrepancy between aggregometric analysis in PRP and in whole blood presumably results from the differences between the methods (turbidimetric vs. impedance method) and the testing in different milieus (PRP vs. citrated whole blood). Interestingly, in PRP, both clinically affected patients showed a markedly diminished response to low but not to the high concentration of collagen in aggregometry, highlighting the importance of using the appropriate agonist concentrations for the detection of platelet dysfunction.

Although certain inherited platelet function defects may be diagnosed by functional testing alone, the majority of cases suspected of an inherited bleeding disorder require further genetic testing. Indeed, the large clinical heterogeneity is reflected by an increasing number of genetic causes that have been implicated in platelet dysfunction. 2 Thus, only broad genetic testing can fully cover the entire range of gene mutations underlying IPFDs. Several other mutations, leading to truncated proteins and a loss of function, have been described until today, four of which were also located in the REM domain. 4 7 For other mutations leading to stop codons, a missing RasGRP2 expression on platelets could be shown. 3 6

The knowledge of the genetic causes of IPFDs is important for counselling issues and enables prenatal testing. Moreover, genetic diagnostics are expected to have a greater impact in the future as novel therapeutic strategies targeting specific genes will become available. 12 Identification of the genetic cause underlying abnormal bleeding has also some impact on prophylactic and therapeutic strategies, although therapeutic options for bleeding control in IPFDs are limited. 13 Therapy of the two patients presented included treatment with the antifibrinolytic tranexamic acid and iron supplementation. Massive menorrhagia required additional hormonal treatment to inhibit ovulation. This represents established treatment strategies in IPFDs. 14

Taken together, our findings expand the genotypic spectrum of RasGRP2 deficiency and highlight the validity of whole exome sequencing as a diagnostic tool for rare inherited bleeding disorders.
