# Supplementary material for: Severe Bleeding Diathesis in Siblings with Platelet Dysfunction due to a Novel Nonsense RASGRP2 Mutation
Source: TH Open. 2020 Dec 25;4(4):e413–6. doi: 10.1055/s-0040-1718910 (PMC7762629; doi:10.1055/s-0040-1718910)
Supplement: Supplementary file 1 — Supplementary Material [file 10-1055-s-0040-1718910-s200018.pdf]

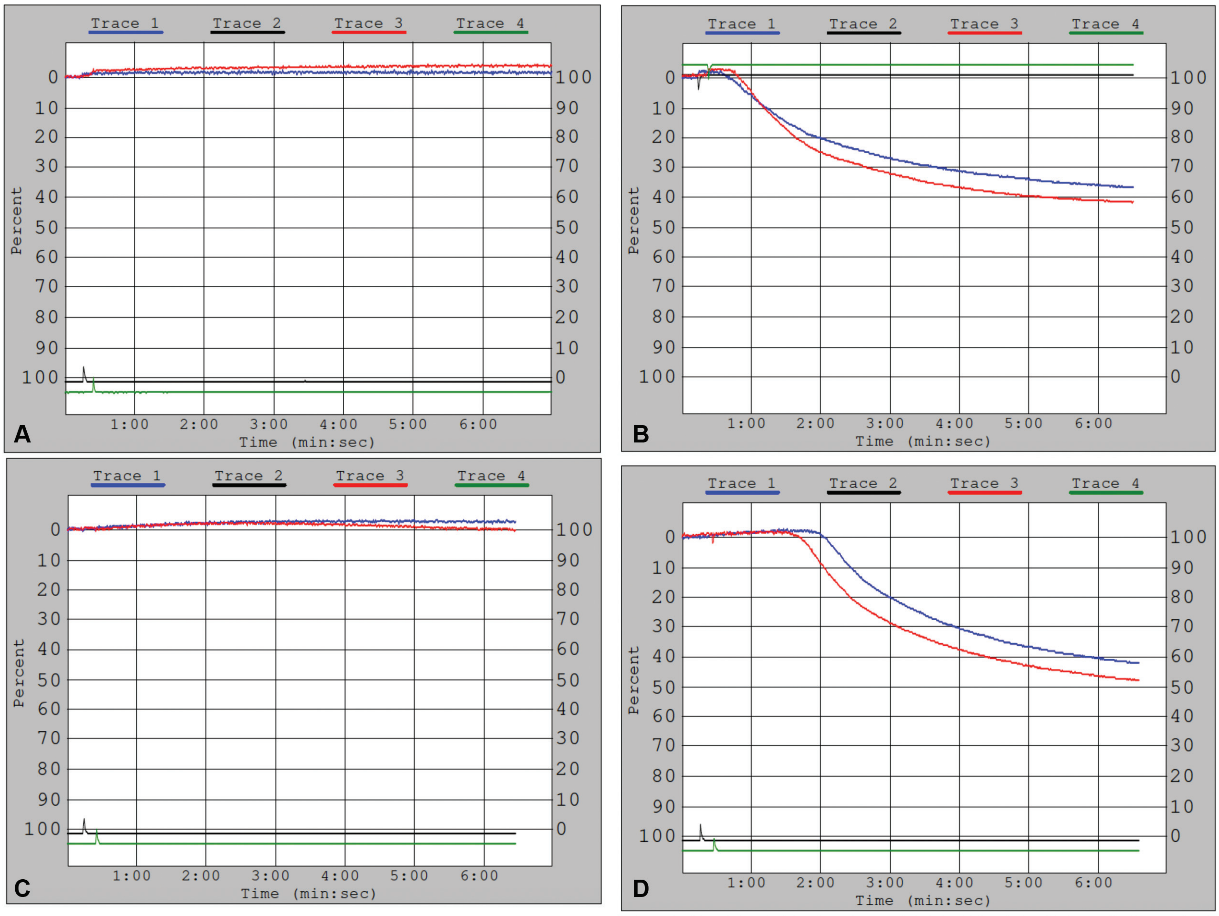

Supplementary Fig. S1 Whole blood aggregometry curves from clinically affected index patient (**A**,**B**; patient 1, homozygous *RASGRP2* mutation) and clinically unaffected heterozygous mutation carrier (**B**,**D**; sister). In the patient affected by a homozygous *RASGRP2* mutation, neither ADP (**A**) nor collagen (**C**) at rather high concentrations induced platelet aggregation. In accordance with the results in PRP, for arachidonic acid, a normal aggregation response was observed in whole blood (curves not shown). The heterozygous mutation carrier shows a normal aggregation pattern with ADP (**B**), collagen (**D**), and arachidonic acid (not shown).
